# Health Tract No. 110

**Published:** 1865

**Authors:** 


HEALTH TRACT No. 110.

### SLEEP AND DEATH.

As men begin to be about fifty years old, especially if of sedentary habits, the feeling on rising in the morning is as if they had not gotten enough sleep, not as much as they used to have, and as if they would like to have more, but they can not get it. They look upon a healthy child sleeping soundly with a feeling of envy. But it is curious to observe that there is a bliss to all in the act of going to sleep, a bliss we become cognizant of only when we happen to be aroused just as we are falling into sound sleep; and there are strong physiological reasons to suppose that this state is a counterpart of that great event which is to come upon all, the act of dying. In fact, those who have in rare cases been brought back to life when on its extremest verge, and in several cases as to those who have been recovered from drowning and other modes of strangulation, or simple smothering, called "asphyxia" by physicians, the expressions have been, on coming to consciousness: "How delicious!" "Why did you not let me go?" An eminent name, thus brought back, represented that the last-remembered sensations of which he was conscious were as if he were listening to the most ravishing strains of music. Let us all then cherish the thought that our approach to the sleep of the grave is the strict counterpart of the approach to sleep, of which some nameless writer has beautifully said: "It is a delicious moment; the feeling that we are safe, that we shall drop gently to sleep. The good is to come, not past. The limbs have been just tired enough to render the remaining in one position delightful, and the labor of the day is done. A gentle failure of the perceptions comes slowly creeping over us; the spirit of consciousness disengages itself more and more, with slow and hushing degrees, like a fond mother detaching her hand from that of her sleeping child; the mind seems to have a balmy lid closing over it, like the eye, closing, more closed, closed altogether! and the mysterious spirit of sleep has gone to take its airy rounds." May such be the physical "bliss of dying" to you and to me, reader, with the spiritual added, ten thousand times more ineffable.

From Dr Hall's book on "Sleep." $1.60. P. P.

